# Interaction of *Neisseria meningitidis* carrier and disease isolates of MenB cc32 and MenW cc22 with epithelial cells of the nasopharyngeal barrier

**DOI:** 10.3389/fcimb.2024.1389527

**Published:** 2024-05-02

**Authors:** Simon Peters, Katherina Mohort, Heike Claus, Christian Stigloher, Alexandra Schubert-Unkmeir

**Affiliations:** ^1^Institute for Hygiene and Microbiology, Julius-Maximilian University Wuerzburg, Wuerzburg, Germany; ^2^Imaging Core Facility, Biocenter, Julius-Maximilian University Wuerzburg, Wuerzburg, Germany

**Keywords:** *Neisseria meningitidis*, clonal complexes, transmigration, air-liquid-interface, epithelial barrier

## Abstract

*Neisseria meningitidis* (Nm, the meningococcus) is considered an asymptomatic colonizer of the upper respiratory tract and a transient member of its microbiome. It is assumed that the spread of *N. meningitidis* into the bloodstream occurs via transcytosis of the nasopharyngeal epithelial barrier without destroying the barrier layer. Here, we used Calu-3 respiratory epithelial cells that were grown under air-liquid-interface conditions to induce formation of pseudostratified layers and mucus production. The number of bacterial localizations in the outer mucus, as well as cellular adhesion, invasion and transmigration of different carrier and disease *N. meningitidis* isolates belonging to MenB:cc32 and MenW:cc22 lineages was assessed. In addition, the effect on barrier integrity and cytokine release was determined. Our findings showed that all strains tested resided primarily in the outer mucus layer after 24 h of infection (>80%). Nonetheless, both MenB:cc32 and MenW:cc22 carrier and disease isolates reached the surface of the epithelial cells and overcame the barrier. Interestingly, we observed a significant difference in the number of bacteria transmigrating the epithelial cell barrier, with the representative disease isolates being more efficient to transmigrate compared to carrier isolates. This could be attributed to the capacity of the disease isolates to invade, however could not be assigned to expression of the outer membrane protein Opc. Moreover, we found that the representative meningococcal isolates tested in this study did not damage the epithelial barrier, as shown by TEER measurement, FITC-dextran permeability assays, and expression of cell-junction components.

## 1 Introduction

*Neisseria meningitidis* (Nm, meningococcus) is a Gram-negative human-specific bacterium. Approximately 10–35% of the human population carry meningococci in the nasopharynx as a part of their transient microbiome without symptoms (Yazdankhah and Caugant, 2004; Caugant and Maiden, 2009; Christensen et al., 2010). In rare cases and by unknown mechanisms, *N. meningitidis* can cross the respiratory epithelial barrier, spread in the bloodstream, and cause a severe clinical picture called invasive meningococcal disease (IMD). Carriage status is a prerequisite for the transmigration of the pathogen from the respiratory tract into the bloodstream and thus the development of IMD.

*N. meningitidis* is classified into 12 serogroups based on the antigenic characteristics of its capsular polysaccharide. The vast majority of IMD cases worldwide are caused by six serogroups of *N. meningitidis*: A, B, C, W, X and Y (Whittaker et al., 2017; Parikh et al., 2020). In developed countries, Serogroups B, C, Y, and W account for the great majority of cases of IMD (https://atlas.ecdc.europa.eu/public/index.aspx?Dataset=27&HealthTopic=36; https://www.cdc.gov/meningococcal/surveillance/index.html). Serogroup B isolates (MenB) clonal complex (cc) 32 is a major hypervirulent lineage, whilst MenW cc22 lineage causes sporadic and endemic outbreaks (Tsang et al., 2016).

The nasopharynx is located posterior to the nasal cavity and is lined with pseudostratified columnar epithelium. The pseudostratified columnar airway epithelium contains ciliated cells, goblet cells and basal cells (respiratory epithelium). The nasopharynx is also covered by stratified squamous epithelium, which comprises about 60% of the nasopharyngeal epithelium. Specialized structures, termed tight junctions, separate the epithelial layer into apical (luminal) and basolateral components, which form an important barrier for the passage of molecules and microbes from the lumen of the airways. In addition to their role in physical barrier function, respiratory epithelial cells are essential for the normal function of the mucociliary clearance. They produce a variety of antimicrobial molecules and contribute to the initiation and regulation of inflammatory responses.

The epithelium of the respiratory tract is coated with mucus, a multi-component secretion, that is produced by goblet cells. Mucus exists in two discreet layers (Zanin et al., 2016): a periciliary liquid layer (PCL), which bathes surface cilia and contacts the surface of the epithelial cells, and a more viscous gel layer on top. The gel layer contains the secreted mucins MUC5AC and MUC5B, while the PCL comprises the membrane-bound mucins MUC1, MUC4 and MUC16 (Zanin et al., 2016). Mucins have been shown to have a direct strong antipathogenic effect. For example, MUC5AC can be upregulated in the intestine during nematode infection and directly reduce ATP levels in the nematodes (Hasnain et al., 2011). MUC2 acts as a chemoattractant; it binds to *Campylobacter jejuni* and has been shown to limit its growth (Tu et al., 2008). Bacteria can, however, employ various strategies to overcome the mucus layer and subsequently reach the epithelial surface of the mucous membrane.

Once *N. meningitidis* reaches the mucosal epithelial surface, the bacterium possesses several factors that promote adhesion to host tissues and biofilm formation. *N. meningitidis* expresses the type IV pili (Tfp), the outer membrane proteins Opa and Opc, and several so-called minor adhesion proteins, that mediate binding to the respective receptors on epithelial cells [reviewed in (Hill et al., 2010)]. Meningococcal Tfp are suggested to mediate attachment to host cells by binding to CD46 (Källström et al., 1997). However, studies with the related species *N. gonorrhoeae* have shown that pilus-mediated binding is not dependent on cellular CD46 expression (Tobiason and Seifert, 2001; Kirchner et al., 2005), but related on binding to integrins shown on urethral epithelial cells (Edwards and Apicella, 2005). Opa proteins mediate binding to CEACAMs (carcinoembryonic antigen-related cell-adhesion molecule) and HSPGs (heparan sulfate proteoglycans), and the Opc protein can interact with HSPGs and, via vitronectin and/or fibronectin, bind to integrin receptors (Virji et al., 1992, 1994, 1995, 1999; Unkmeir et al., 2002; Sa E. Cunha et al., 2010). The Opc protein is encoded by a single gene, *opcA*, which is only expressed in *N. meningitidis* but not in the related species *N. gonorrhoeae*. Opc is a β-barrel protein, with five surface-exposed loops and its crystal structure was elucidated in 2002 (Prince et al., 2002). Expression of Opc is due to phase variation via slipped strand mispairing within a poly-cytosine tract in the promoter region of *opcA* (Sarkari et al., 1994). The number of cytosine bases in this region controls the level of expression of Opc protein: if the number of cytosines is less than 10 or higher than 15, Opc is not expressed (Sarkari et al., 1994). While the bacterial factors that mediate binding to epithelial cells have been well studied and characterized, the mechanism of passage across the epithelial cell barrier has long been controversial (Birkness et al., 1995; Pujol et al., 1997). A study by Sutherland and colleagues published in 2010, using Calu-3 cells, demonstrated that meningococci cross the epithelial cell barrier by a transcellular route; when crossing the layer, its integrity was not disrupted and the bacteria were detected within the cells of the monolayer (Sutherland et al., 2010). Furthermore, the authors were able to show that the successful crossing of the epithelial cell barrier by *N. meningitidis* requires the expression of Tfp and capsule and is dependent on the microtubule network of the host cell. In this study, Calu-3 cells were maintained under liquid-liquid interface (LLI) cell culture conditions. The study by Audry et al., used an air-liquid-interface (ALI) culture to allow formation of pseudostratified layers and mucus production, and showed that meningococci tend to reside in the mucus of the Calu-3 cell layer (Audry et al., 2019).

The respiratory mucus represents a hitherto little-studied factor in the role of *N. meningitidis*-host interactions. Therefore, determining the interaction of *N. meningitidis* with mucus-producing epithelial cells of the respiratory tract and the mechanisms of passage through the epithelial layer is important for understanding both meningococcal carriage and disease. In this study we applied representative isolates of MenB:cc32 and MenW:cc22, including disease and carrier isolates, to study their interaction with the human bronchial epithelial cell line Calu-3, that can differentiate into polarized monolayers when grown on porous membranes *in vitro* (Foster et al., 2000). Moreover, Calu-3 cells were grown under ALI conditions to induce formation of pseudostratified layers and production of mucus (Kreft et al., 2015). The rate of bacterial localization in the outer mucus, as well as the number of cell-associated bacteria, cellular adhesion, invasion and transmigration, the impact on barrier integrity and cytokine release was assessed.

## 2 Materials and methods

### 2.1 Bacterial strains and growth conditions

Bacteria were grown overnight on Columbia agar plates with 5% sheep blood (bioMérieux) at 37°C in 5% CO_2_. On the next day, a fraction of the bacteria was transferred to 10 ml proteose peptone medium (PPM) supplemented with 1× Kellogg´s supplement, 0.01 M MgCl_2_, and 0.005 M NaHCO_3_ (PPM+) and incubated for 90 min at 37°C in an orbital shaker at 200 rpm. *Neisseria meningitidis* strain MC58 is a serogroup (Sg) B (MenB) isolate (United Kingdom, 1983) of the clonal complex (cc) 32 and was kindly provided by E. R. Moxon (Tettelin et al., 2001). *N. meningitidis* Sg C (MenC) strain 8013/clone12 (cc18) was kindly provided by M. Taha (Nassif et al., 1993). *N. meningitidis* SgB (MenB) strain α711 (cc32) and *N. meningitidis* SgW (MenW) strain α275 were isolated during the Bavarian meningococcal carriage study (Claus et al., 2005; Harrison et al., 2013). *N. meningitidis* SgW (MenW) strain DE13664 was isolated from CSF. Meningococcal strains DE13664 and α711 were subject to whole-genome sequencing. Genome sequencing was performed on an Illumina NextSeq 500 sequencer (Illumina Inc., San Diego, CA, USA) at the core unit Systems Medicine of the University of Würzburg. The resulting FASTQ files were *de novo* assembled using the Velvet assembler integrated in Ridom SeqSphere+ software (Ridom GmbH, Münster, Germany). All strains are summarized in **Table 1**.

**Table 1:** Strains used in the study.

strain	8013	MC58	α711	DE13664	α275
PubMLST ID	1038	240	10183	84878	9756
*Molecular Typing*					
Serogroup (% of carriage/cases)*	C (3.3/17.2)	B (26.8/54.6)	B (26.8/54.6)	W (6.4/10.3)	W (6.4/10.3)
*Sequence type (ST)*	ST-177	ST-74	ST-32	ST-22	ST-22
*Clonal complex (CC)*	ST-18	ST-32	ST-32	ST-22	ST-22
*porA-VR1*	21	7	7	18-1	18-1
*porA-VR2*	26-2	16-2	16	3	3
*fetA*	F1-5	F1-5	F3-3	F4-1	F4-1
*General information*					
*No. of contigs (>2 kb)*	1	1	228	158	133
*Genome size, bp*	2,277,550	2,272,360	2,113,949	2,150,017	2,242,661
*G+C content, %*	51.4	51.5	51.75	51.5	51.2
*GenBank accession no.*	FM999788	AE002098	NA	NA	AM889138
Epidemiology*					
*Source of the isolate*	patient	patient	carrier	patient	carrier
*Frequency of ST in carriers (%)^+^*	0	0.05	1.0	2.5	2.5
*Frequency of CC in carriers (%)^+^*	0.7	6.0	6.0	8.6	8.6
*Frequency of ST in cases (%)^+^*	<0.01	0.05	3.3	0.5	0.5
*Phenotypic characterization*					
*Opa expression*	–	+	+	+	+
*Opc expression*	–	+	–	–	+
*Class I type IV pilus*	+	+	+	+	+

^*^Based on all isolates (43,336) in Europe with an assigned disease/carrier status listed in PubMLST (2023-12).

^+^Based on all carrier (15,432) or case isolates (27.904) in Europe listed in PubMLST.

^#^Based on case isolates with assigned disease for each ST or CC in Europe listed in PubMLST.

ND, Not determined; NA, Not available.

### 2.2 Cell culture

The human lung epithelial cell line Calu-3 (ATCC: HTB-55) was routinely cultivated in A-MEM (Advanced Minimum Essential Medium, Gibco) supplemented with 4 mM GlutaMAX (Gibco) and 2,5% fetal bovine serum (FBS; Thermo Fisher) based on the described optimized cell culturing conditions for Calu-3 cells in the study by Kreft et al. (Kreft et al., 2015). Cells were maintained at 37° C and 5% CO_2_ and 95% relative humidity until they reached 80-100% confluence. The cell culture medium was changed three times per week. For the development of Calu-3 cell models on the air-liquid or liquid-liquid interfaces, the cells were seeded on polyethylene terephthalate (PET) porous membranes with pore sizes of 3 µm (24-Well inserts, Greiner) and a seeding density of 4×10^5^ cells/cm^2^ following the protocols by Kreft et al. (Kreft et al., 2015). For infection experiments, permeable cell culture inserts (12-, or 24-Well inserts, Greiner), with a pore size of 3 µm were used and coated with 0.2% gelatin for 1 h at 37°C. Afterwards, 800 µl (for 24-Well inserts) or 1600 µl of medium (for 12-Well inserts) was added to the basolateral chamber and cells were seeded in 200 µl (for 24-Well inserts) or 600 µl (for 12-Well inserts) medium on the apical side at a density of 4×10^5^ cells/cm^2^. Cells were allowed to grow for seven days in a humidity incubator, at 37°C and 5% CO_2_, followed either by the removal (air lift) (for air-liquid interface (ALI) conditions) or replacement (for LLI conditions) of the apical, and replacement of the basal medium. Cells were grown for further seven days and barrier tightness was assessed by measuring the trans-epithelial electrical resistance (TEER) (Ω cm^2^) prior to the experiment. To assess the integrity of the barrier under ALI culture conditions, 200 µl of medium was placed on the apical side of the cells for 1 h, followed by the TEER measurement and then the medium was carefully removed again after the measurement.

### 2.3 Infection assay

(i) For infection experiments, bacteria were cultured as noted above. Cells were infected with 1x10^4^ (24-Well inserts) or 4x10^4^ (12-Well inserts) bacteria for 24 h at 37°C and 5% CO_2_, respectively. Calu-3 cells grown under ALI conditions were infected on the apical side by carefully applying the suspension of bacteria in 10 µl (24-Well inserts) or 40 µl (12-Well inserts). Calu-3 cells grown under LLI conditions, were infected with a suspension of bacteria in 200 µl, which was added to the medium on the apical side.

(ii) Adherence and invasion. The number of adherent and invasive bacteria was determined as described previously (Peters et al., 2019; Endres et al., 2022). Briefly, cells grown under ALI or LLI culture conditions were infected for 24 h with the indicated strains using the CFUs described above. After infection, the apical sides of the cells were washed three times with 200 µl PBS and the Transwell™ membrane inserts were transferred into empty 24-Well plates. Cells were treated with 300 µl 1% saponin for 15 min and scraped off to determine the total number of adherent bacteria. To determine the number of invasive bacteria, Transwell™ membrane inserts were transferred, after the washing steps, into fresh medium containing 200 µg/ml gentamicin and 200 µl gentamicin containing medium was added to the apical side. After 2 h incubation at 37°C, Transwell™ membrane inserts were washed again and further processed as described recently (Peters et al., 2019; Endres et al., 2022). To separate the bacteria in the outer mucus fraction from the cell-associated fraction, infected cell layers were incubated for 15 min with 200 µl of A-MEM-0.1% N-acetyl-L-Cysteine (N-ACC, Carl Roth) and harvested. This step was repeated three times as described by Audrey et al. (Audry et al., 2019). CFU were determined by plating serial dilutions. N-ACC treatment did not affect bacterial growth (**Supplementary Figure S1**). Cells were furthermore treated with saponin as described for the total adherence, scraped off and bacterial numbers were assessed by plating serial dilutions to determine CFU to assess the number of cell-associated bacteria.

(iii) Transmigration assay. Bacterial transmigration was determined as recently described (Martins Gomes et al., 2019; Endres et al., 2022). The cells were infected with the indicated bacterial isolate for 24 h. The inserts were then washed three times with PBS and transferred to wells of a 24-Well plate filled with 800 µl of fresh culture medium. Bacteria were allowed to transmigrate for 1 h and samples were taken for plating in a dilution series from the basolateral medium to determine bacterial CFU.

### 2.4 TEER determination and permeability assays

Barrier tightness was assessed by measuring the transepithelial electrical resistance (TEER) using a Millicell^®^ ERS-2 Volt-Ohm Meter (Merck). In addition, the integrity of the cell monolayer was assessed using 70 kDa fluorescein isothiocyanate (FITC)-dextran (Sigma) permeability assays or sodium fluorescein (NaF) permeability measurements as described previously with minor adaptations (Martins Gomes et al., 2019; Endres et al., 2022). Briefly, 200 µl of medium with 10 µM NaF or 1 mg/ml FITC-Dextran were added to the apical side of infected or non-infected cells and samples were taken every 15 min for a total of 1 h from the basolateral chamber. The samples were analyzed using a fluorescence plate reader (Infinite Pro, Tecan) and the permeability was calculated as described previously (Stebbins et al., 2016). TEER values of more than 400 Ω cm^2^ were considered to present a monolayer with intact tight junctions as described recently (Min et al., 2016).

### 2.5 Immunofluorescence

For immunofluorescence imaging, Calu-3 cells were grown under LLI or ALI culture conditions as described above. Inserts were fixed with 2% formaldehyde and 0.2% glutaraldehyde (GA) for 15 min at room temperature, washed twice with PBS and permeabilized for 15 min with PBS containing 0.1% saponin and 2% bovine serum albumin (BSA) (staining buffer). The cells were immunolabeled with primary antibodies against MUC5AC (RRID: AB_10978001, clone 45M1; 1:250 in staining buffer), CRB3 (RRID: AB_2640142; 1:200 in staining buffer) for 90 min, washed three times and stained with Alexa Fluor (AF) conjugated secondary antibodies (Molecular Probes, Invitrogen, Thermo Fisher Scientific) for 45 min. After three washing steps with PBS, the cytoskeleton was stained using Phalloidin-488 for 15 min at 4°C and the nuclei were stained with Hoechst33342 for 15 min at 4° C. Insert membrane was then cut out and mounted with FluoroShield (Sigma-Aldrich) on glass slides. The membrane was covered with a high precision cover glass and imaging was performed on a Nikon Ti Eclipse spinning disc microscope. Image analyses were performed using the NIS-Elements software (Nikon) and FIJI ImageJ version 1.52 (NIH). For ZO-1 and occludin staining we followed a protocol provided by STEMCELL Technologies (https://www.stemcell.com/technical-resources/educational-materials/protocols/how-to-perform-immunocytochemistry-icc-staining-of-epithelial-cells-cultured-as-monolayers-or-at-the-air-liquid-interface.html). Briefly, Calu-3 cells were infected with 1x10^4^ bacteria for 24 h. The infected cells on Transwell™ inserts were washed three times with PBS and then fixed overnight in ice-cold methanol at -20°C. The following day, the lower part of the inserts was washed with ice-cold acetone for 1 min. To permeabilize the samples, incubated on a shaker in staining buffer [1% BSA containing Triton X-100 (Roth 3051.4) and Tween 20 (Roth 9127.1)] for 1 h at room temperature. Afterwards, the inserts were washed three times with PBS. To detect tight junction proteins, the samples were incubated overnight at 4°C with primary antibodies against ZO-1 (Proteintech 2-1773-1-AP)) and occludin (BD Transduction Laboratories 611091). The next day, the inserts were washed in PBS and secondary staining was performed for 1 h at room temperature. Therefore, secondary antibodies Alexa Fluor 488 goat anti-mouse (Invitrogen) and Alexa Fluor 555 donkey anti-rabbit (Invitrogen) at a dilution of 1:200 in 1% BSA were used. Subsequently DAPI staining was performed (Invitrogen D1306; 1:1000 in PBS). The Transwell™ membranes were then cut out, mounted on glass slides (mounting medium: Fluoroshield, Sigma) and incubated at 4°C overnight. The imaging was performed on a Nikon Ti Eclipse spinning disc microscope. Image analyses were performed using the NIS-Elements software (Nikon).

### 2.6 Transmission electron microscopy

Preparation of the samples were performed as described previously (Endres et al., 2022). Briefly, Calu-3 cells were infected with 1x10^4^ bacteria for 24 h and afterwards fixed over night with 2,5% GA at 4°C. On the next day, samples were rinsed five times with cacodylate buffer pH 7,2 followed by the incubation with 2% osmium tetroxide for 90 min. The samples were than washed five times with ddH_2_O and the membranes were cut out of the inserts. Following, membranes were contrasted with 0,5% watersoluted uranyl acetate overnight. Before dehydration of the samples, membranes were washed five times with ddH_2_O and dehydration was performed through the addition of ethanol solutions with ascending concentrations (50%, 70%, 90%, 3 × 100%, each time for 30 min.) followed by 30 min exposure to propylenoxid. Embedding was conducted using a 1:1 solution of propylenoxid and durcopan which was added to the samples and allowed to dry overnight. Subsequently the samples were immersed in pure durcupan, which was changed twice each two h. Finally, the samples were placed in an embedding capsule polymerized at 60°C for 48 h, ultrathin sectioned and contrasted. The sections were examined by a JEM-1400Flash transmission electron microscope (JEOL) equipped with a Matataki camera system.

### 2.7 Quantitative PCR

The expression of tight junction proteins and cytokines was determined using quantitative PCR (qPCR). Calu-3 cells were grown on 12-Well inserts under ALI or LLI culture conditions, respectively, and were infected for 24 h with 4x10^4^ bacteria. The cells were then lysed, and the RNA purified using the NucleoSpin RNA kit (Macherey-Nagel) according to the manufacturer’s instructions. RNA concentration was determined with the NanoDrop (Thermo-Fisher), and 500 ng were used for cDNA synthesis using LunaScript RT (New England Biolabs). qPCR was conducted on a StepOnePlus real-time PCR Thermocycler (Applied Biosystems) using PowerUp SYBR Green master mix (Applied Biosystems). Primer sequences are listed in **Table 2**. All primers were validated by primer efficiency analysis and specificity was controlled by agarose gel electrophorese of the qPCR products. All data are presented as fold change over 18S rRNA using the cycle threshold (ΔΔCt) calculation (Livak and Schmittgen, 2001).

**Table 2:** Primers used for qPCR.

Gene	Forward sequence	Reverse sequence
*18S rRNA^a^*	GTAACCCGTTGAACCCCATT	CCATCCAATCGGTAGTAGCG
*OCLN*	ATGGCAAAGTGAATGACAAGCGG	CTGTAACGAGGCTGCCTGAAGT
*CXCL8^c^*	AGCTCTGTGTGAAGGTGCAG	AATTTCTGTGTTGGCGCAGT
*CXCL1^c^*	CTCTTCCGCTCCTCTCACAG	GGGGACTTCACGTTCACACT
*CXCL2^c^*	CTCAAGAATGGGCAGAAAGC	AAACACATTAGGCGCAATCC
*IL6^c^*	GGAGACTTGCCTGGTGAAAA	CAGGGGTGGTTATTGCATCT
*CCL20^c^*	GCGCAAATCCAAAACAGACT	CAAGTCCAGTGAGGCACAAA
*TJP1*	GTCCAGAATCTCGGAAAAGTGCC	CTTTCAGCGCACCATACCAACC

^a^ (Rho et al., 2010), ^b^ (Kim et al., 2019), ^c^ (van Sorge et al., 2008).

### 2.8 IL-8 and CCL20 ELISA

Samples for ELISA were collected from the basolateral chamber of Calu-3 cells grown on 12-Well inserts under ALI or LLI culture condition after 24 h of infection with 4x10^4^ bacteria of the indicated strains. For detection of IL-8, the Human IL-8 ELISA Set (BD Biosciences) was used according to the manufactures protocol and as described previously (Endres et al., 2022). For detection of CCL-20, the commercially available ELISA was used (R&D Systems). The ELISA was performed according to the manufacture’s instruction using the reagents provided within the kit.

### 2.9 Statistics

Statistical analysis was conducted using the GraphPad Prism software (version 6.01, GraphPad Software Inc.). For pairwise comparison, a two-tailed Student´s t test was used, and analysis of variance (ANOVA) followed by Dunnett’s multiple comparison test was used for multiple comparisons. Statistical significance was accepted at a P value below 0.05.

## 3 Results

### 3.1 Interaction between Calu-3 epithelial cells grown at an air-liquid interface or a liquid-liquid interface and *N. meningitidis*

**Figure 1:**
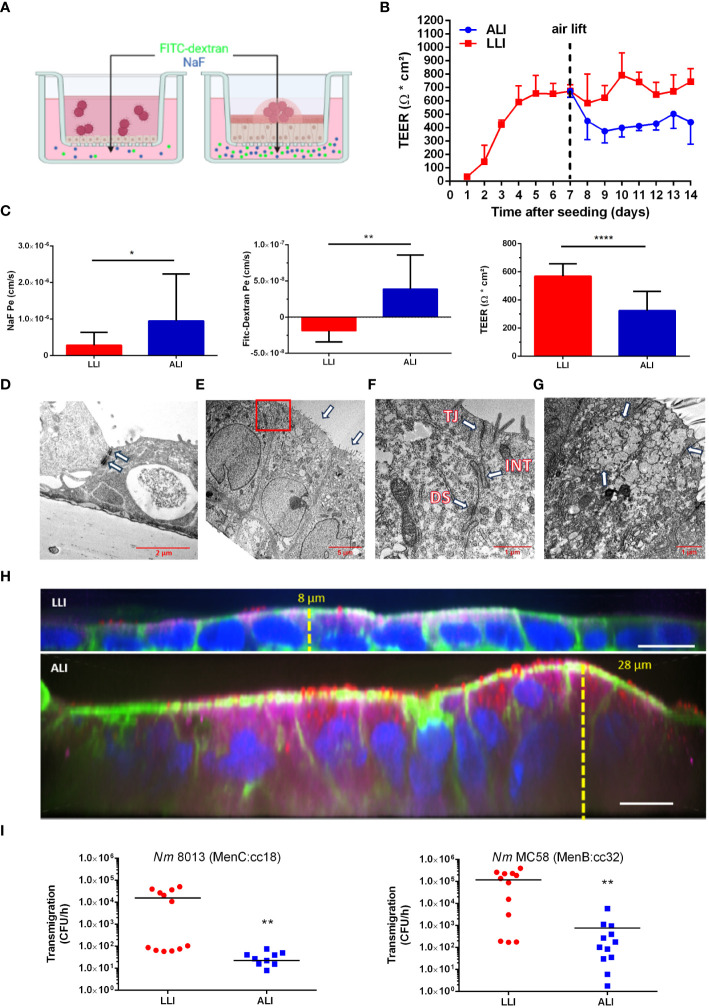
Comparison of the interaction of *N. meningitidis* with Calu-3 cells grown under liquid-liquid interface (LLI) or air-liquid interface (ALI) conditions. **(A)** Schematic representation of Calu-3 cells grown either under LLI or ALI cell culture conditions and infected with *N. meningitidis*. Figure created with BioRender.com. **(B)** Transepithelial electrical resistance (TEER) of Calu-3 cells cultured under LLI or ALI cell culture conditions. TEER values were measured daily after seeding over a period of 14 days. On day seven, medium was removed from the apical side of the insert immediately after TEER readings to establish the ALI condition (air lift). **(C)** Sodium fluorescein (NaF Pe), FITC-Dextran (70kDa) permeability, or TEER values, performed on day seven after air lift or day 14 of LLI culture, respectively. **(D–G)** Transmission electron microscopy (TEM) of Calu-3 cells grown under LLI **(D)** or ALI **(E–G)** conditions. **(D)** Calu-3 cells grown under LLI conditions show a cuboidal monolayer with cell junctions (white arrow). **(E)** Calu-3 cells grown under ALI conditions show a pseudostratified columnar epithelium. Nuclei are located on the basolateral side of cells demonstrating apical-basal polarization. On the apical side of the cells are well developed microvilli (white arrows). The boxed region in panel E indicates the section enlarged in **(F)**. **(F)** Enlargement of the region between cells grown under ALI revealed the presence of large cell junctions. Depicted are the tight junctions (TJ), directly beneath the microvilli, interdigitations (INT), and desmosomes (DS). **(G)** Mucus-producing cell grown under ALI conditions with an abundance of mucin vesicles (white arrows). **(H)** Immunofluorescence staining of Calu-3 cells grown under LLI or ALI conditions. Cells were stained for cytoskeletal actin filaments with phalloidin (green), for mucus [mouse anti-MUC5AC (red)], for cell polarity [anti-CRB 3 (magenta)], or nuclei [Hoechst 33342 (blue)]. Scale bars represent 10 µm. **(I)** *N. meningitidis* interaction with Calu-3 cells grown under either LLI or ALI conditions. Cells were infected with 1x10^4^ bacteria from the apical side with either MenC:cc18 strain 8013 or MenB:cc32 strain MC58 for 24 h. *N. meningitidis* transmigration rates were determined by enumeration of CFU in the basolateral compartment after 1 h of incubation in fresh basolateral media. Results show the mean ± SD from three independent experiments. *P < 0.05; **P < 0.001; ****P < 0.0001; unpaired, two-tailed Student’s t-test.

A unique feature of the respiratory epithelium is that the apical side of the cells faces the air. To mimic this under laboratory conditions, we used the Calu-3 human lung epithelial cell line (ATCC HTB-55), cultured the cells at an air-liquid interface (ALI), and compared it with the culture model at the liquid-liquid interface (LLI) (**Figures 1A–I**). Calu-3 cells were grown on Transwell™ inserts with a 3 µm pore membrane in A-MEM supplemented with GlutaMAX and FBS in the apical and basal compartment. The cultures were maintained for 7 days until a transepithelial electrical resistance (TEER) ≥ 700 Ω cm^2^ was obtained, corresponding to a confluent culture. Calu-3 differentiation was initiated by changing culture conditions to ALI by removing the apical medium, whereas for LLI media from the apical and basal chamber were replaced with fresh media. The formation of the epithelial barrier was next monitored by measuring the TEER for further 7 days (**Figure 1B**). We observed an increase of the TEER with a maximum value of 744.7 ± 93.3 Ω cm^2^ at day 14 at LLI conditions (**Figure 1B**). TEER values of cells at the ALI conditions were approximately 40% below the values at the LLI condition. At day 8 (day 1 after air lift), the TEER decreased to 450.2 ± 151.5 Ω cm^2^, however, a TEER > 400 Ω cm^2^ was maintained for at least 7 days after establishing ALI conditions (**Figure 1B**). The paracellular permeability of the Calu-3 cells was measured by permeability assays on day 14 to confirm the TEER results (**Figure 1C**). Permeability to sodium fluorescein (NaF) was measured at 9.5 ± 12.6 × 10^–7^ cm/s for cells grown under ALI culture conditions, while values of 2.8 ± 3.4 × 10^–7^ cm/s were observed for cells grown under LLI culture conditions (**Figure 1C**). In parallel, permeability for fluorescein isothiocyanate (FITC-)dextran (70kDa) was determined and assessed at 3.9 ± 4.6 × 10^–8^ cm/s for cells grown under ALI culture conditions, while values of -1.8 ± 1.4 × 10^–8^ cm/s were observed for cells grown under LLI culture.

Calu-3 cells grown at the ALI and LLI culture conditions were then characterized using transmission electron microscopy (TEM) and confocal immunofluorescence imaging. Using TEM, we observed various electron dense regions between adjacent cells most likely representing cellular junctions including tight junctions or adherens junctions when cells were grown at LLI conditions (**Figure 1D**). Calu-3 cells grown under ALI culture conditions showed a pseudostratified columnar epithelium (**Figure 1E**). Moreover, cells showed apical-basal polarization with nuclei located on the basolateral side of cells and microvilli formation at the apical surface (**Figure 1E**). Tight junctions and adhesion belts were present at sites of cell-cell contact (**Figure 1F**). Calu-3 cells differentiate into ciliated and non-ciliated cells. The latter produce mucus and have previously been described as goblet-like cells (Shen et al., 1994; Kreft et al., 2015). A representative image shows a mucus-producing cell with an abundance of mucin vesicles (**Figure 1G**). Cells were next stained for F-actin with phalloidin (green), for nuclei with Hoechst (blue), for secreted mucin with anti-MUC5AC (red) antibody, and for cell polarity with anti-crumbs cell polarity complex component (CRB) 3 (magenta) antibody and analyzed by confocal microscopy (**Figure 1H**; **Supplementary Figure S2**). Calu-3 cells grown at LLI conditions showed a densely packed and thin monolayer, approximately 8 µm high, with low expression of mucin and CRB3. In contrast, Calu-3 cells grown at ALI conditions showed a stratified columnar epithelium with a multicellular columnar cell layer, approximately 20-30 µm in height, with mucus-secreting cells, and strong expression of CRB3 (**Figure 1H**).

We next evaluated the traversal of *N. meningitidis* of Calu-3 cells grown either at ALI or LLI conditions. *N. meningitidis* strain 8013 (MenC:cc18), recently used in the report of Audry et al. (Audry et al., 2019), served as a control. Both models were infected with *N. meningitidis* 8013 or *N. meningitidis* MC58 (MenB:cc32) (characterized in **Table 1**) for 24 h to determine the ability of the bacteria to cross the epithelial barrier (**Figure 1I**). *N. meningitidis* strain MC58 was chosen as a prototype sequence type (ST)-32 strain belonging to the hyperinvasive lineage ST-32 clonal complex (cc). The results showed a significant reduction in the number of transmigrating bacteria for both meningococcal strains tested when Calu-3 cells were grown under ALI conditions compared to cells grown under LLI conditions. Data received for strain *N. meningitidis* 8013 confirmed previously published data using Calu-3 cells grown under ALI culture conditions (Audry et al., 2019).

### 3.2 Carrier and disease isolates of MenB:cc32 and MenW:cc22 primarily colonize the outer mucus of epithelial cells

The number of bacteria found in lower chamber of the Transwell™ were significantly lower when cells were grown under ALI compared to LLI conditions, regardless of which isolate was analysed. One reason might be that bacteria are trapped within the mucus and recent work has indicated, that *N. meningitidis* primarily reside in the outer mucus when cells are grown under ALI conditions, which indicates that the mucus layer is probably the actual niche for the pathogen (Audry et al., 2019). Therefore, we next assessed the ability of *N. meningitidis* isolates to interact with epithelial cells and measured the number of cell-attached bacteria and bacteria found in the outer mucus after 24 h incubation of Calu-3 cells grown under ALI culture conditions. Moreover, we included disease and carrier isolates of two hypervirulent lineages. Isolate α711 was chosen as a representative carrier isolate highly related to MC58 from the ST-32 cc. In addition, a representative disease-carrier pair of MenW:cc22 (DE13664 and α275) was included in the analyses. Although the disease-carrier isolates belong to the same cc, they differ in the expression of some of their outer membrane proteins, for instance in the expression of the major adhesin Opc, which was expressed only by strain MC58 and carrier isolate α275 (**Supplementary Figure S3**) and was not expressed by strain 8013 due to deletion of the *opcA* gene or by the isolates α711 and DE13664 because of phase variation (**Supplementary Figure S3**).

**Figure 2:**
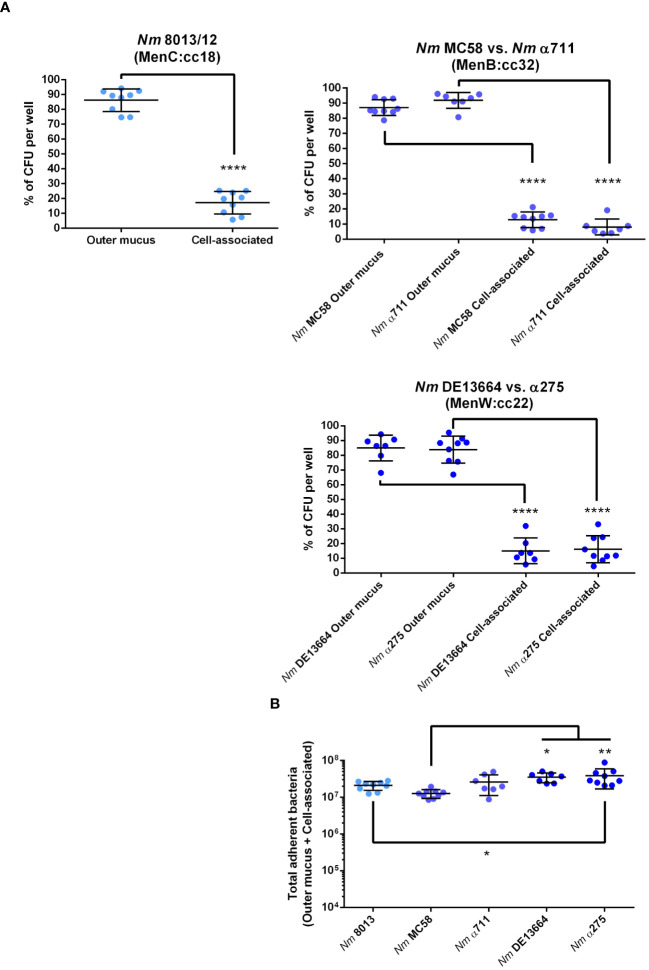
*N. meningitidis* primarily colonizes the outer mucus layer. Calu-3 cells were grown under ALI conditions and infected with 1x10^4^ bacteria of MenC:cc18, MenB:cc32 or MenW:cc22 lineages for 24 h. **(A)** Bacteria associated with the outer mucus layer were collected by N-acetylcysteine treatment. Bacterial fraction from the cell-attached mucus was collected by saponin treatment of the cells. Bacterial loads in both fractions were determined by enumeration of the CFU and are presented as a percentage of CFU ± SD. **(B)** determination of the bacterial loads in both fractions (outer mucus and cell-attached mucus) after 24 h of infection The total number of CFU ± SD is given. All experiments were performed at least three times in triplicates. *P < 0.05; **P < 0.01; ****P < 0.0001; One-way ANOVA.

Following previous studies performed with meningococcal strain 8013 (Audry et al., 2019), we first examined the localization of the bacteria in the mucus layer, which can be separated into cell-associated fraction and outer mucus fractions. After infecting the cells with 1x10^4^ bacteria for 24 h, the outer and cell-attached mucus fractions were collected, and the CFU per sample was determined. Meningococcal strain 8013 showed that the bacteria primarily resided in the outer mucus layer (>80%) as reported previously (Audry et al., 2019) (**Figure 2A**). Similar data were observed for MenB:cc32 and MenW:cc22 isolates (**Figure 2A**). Most bacteria were found in the outer mucus (87.07% for MC58, 91.84% for α711, 84.94% for DE13664 and 83.83% for α275), whereas a lower number of bacteria was found in the cell-attached mucus (12.92% for MC58, 8.15% for α711, 15.05% for DE13664 and 16.16% for α275). In addition, we determined the absolute number of total adherent bacteria (outer mucus and cell-attached bacteria): at 24h post-infection (p.i.) the rate of total adherent bacteria was comparable high for the MenB:cc32 isolates (MC35 and α711) compared to strain 8013 (**Figure 2B**), whereas a slight increase of the number of total adherent bacteria was detected at 24h p.i. for both MenW:cc22 isolates DE13664 and α275 compared to MenB:cc32 isolate MC58. The total number of adherent bacteria for α711 did not significantly differ from the number determined for the MenW:cc22 isolates (**Figure 2B**).

### 3.3 Carrier and disease isolates of MenB:cc32 and MenW:cc22 differ in their ability to overcome the nasopharyngeal epithelial barrier

**Figure 3:**
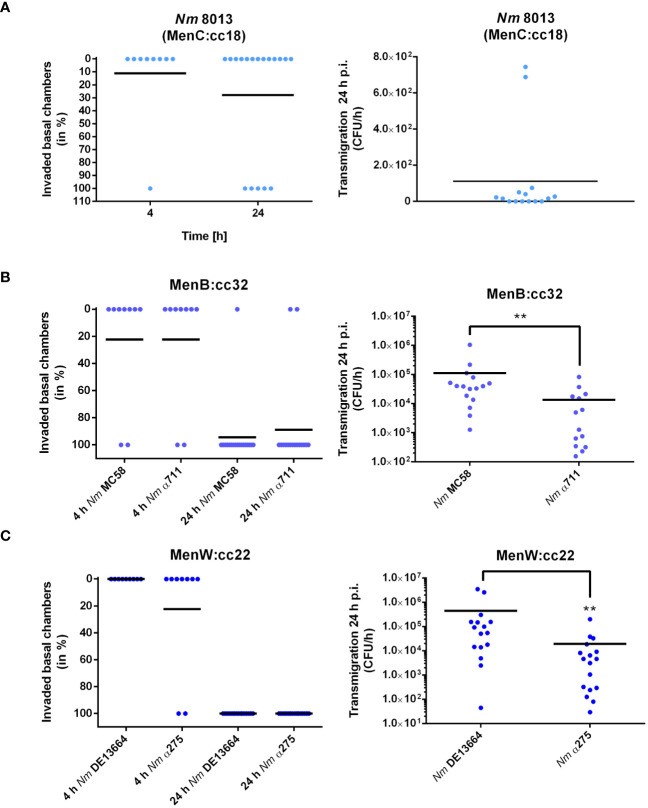
*N. meningitidis* traversing Calu-3 cells grown under ALI conditions. Cells were infected with 1x10^4^ bacteria from the apical side with either MenC:cc18 strain 8013, or representative strains from MenB:cc32 or MenW:cc22 lineages for 24 h. [**(A–C)**, left panels] data are presented as percentage of invaded basal chambers determined from at least nine experiments for 4 h p.i or 18 experiments at 24 h p.i. as described in the study by Audry et al. (Audry et al., 2019). [**(A–C)**, Right panels). *N. meningitidis* transmigration rates were determined by enumeration of CFU in the basolateral compartment after 1 h of incubation in fresh basolateral media. Results show the mean ± SD from three independent experiments. **P < 0.01; unpaired, two-tailed Student’s t-test.

We next determined the frequency of transmigration events for each strain after 4 and 24 h of infection with 1x10^4^ bacteria. Transmigration was analysed by plating the entire amount (for the 4 h timepoint), or appropriate dilutions (for the 24 h timepoint) of the basal media on agar plates. Transmigration was assessed positive if at least one CFU was recovered from the basal chamber as described recently (Audry et al., 2019). When we infected the cells with the MenC:cc18 strain 8013 only 1 out of 9 basal chambers were found positive at 4 h p.i., and 5 out of 18 basal chambers were found positive at 24 h p.i., clearly showing that this strain is mainly non-transmigrating after 4 h of infection, and the frequency just rises insignificantly after 24 h of infection (from 11 to 27%, **Figure 3A**). When we compared disease and carrier isolates of MenB:cc32 or MenW:cc22, we did not detect any differences between MenB:cc32 disease strain MC58 and carrier strain α711 or MenW:cc22 disease strain DE13664 and carrier α275 in their transmigration frequencies (2 out of 9 (4 h) and 17 out of 18 (24 h) basal chambers for MC58, 2 out of 9 (4 h) and 16 out of 18 (24 h) basal chambers for α711, 0 out of 9 (4 h) and 18 out of 18 (24 h) basal chambers for DE13664, 2 out of 9 (4 h) and 18 out of 18 (24 h) basal chambers for α275) (**Figures 3B, C**, left panel). In addition, we examined the absolute number of bacteria that migrated through the barrier. Therefore, the infected chambers were washed and transferred to fresh medium after 24 h of infection, and the number of transmigrating bacteria was counted after 1 h. The results showed that even if the transmigration frequency did not differ between disease and carrier isolates, the total number of bacteria entering the basal chamber was reduced by 80% for α711 compared to MC58 and 95% for α275 compared to DE13664 (**Figures 3B, C**, right panel).

**Figure 4:**
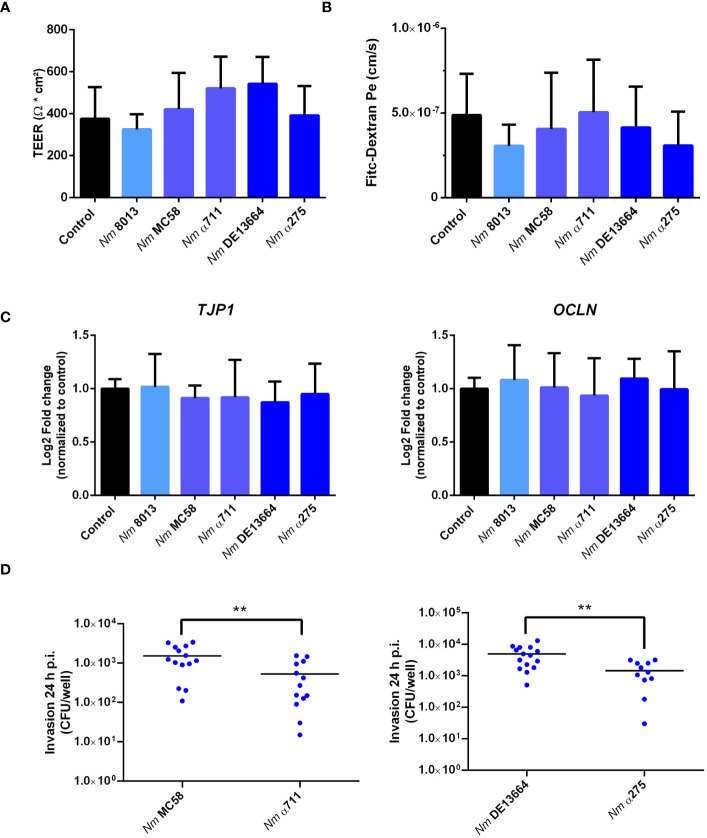
Effects of *N. meningitidis* infection on cell-junction expression in Calu-3 cells, FITC-dextran permeability, or TEER. Calu-3 cells were infected with either MenC:cc18 strain 8013, or representative strains from MenB:cc32 or MenW:cc22 lineages for 24 h **(A)** TEER values of infected and uninfected (control) Calu-3 cells measured at 24 p.i. Data presented as mean ± SD from three independent experiments performed in duplicates. **(B)** FITC-Dextran (70kDa) permeability. Data presented as mean ± SD from three independent experiments performed in duplicates. **(C)** Relative gene expression of tight junction components ZO-1 (TJP1) or occludin (OCLN) quantified by qPCR. Data were normalized to 18S rRNA and presented as the mean Log2 fold change ± SD of three independent experiments performed in duplicates. **(D)** Enumeration of intracellular CFU per monolayer in Calu-3 cells grown under ALI conditions after infection with 1x10^4^ bacteria of the indicated *N. meningitidis* strains, determined by gentamicin protection assays. **P < 0.01; unpaired, two-tailed Student’s t-test.

The number of bacteria detected in the lower chamber of the Transwell™ might result from loss of barrier integrity. In previous research, it has been a question of debate, whether *N. meningitidis* crosses the epithelium by a transcellular (Pujol et al., 1997; Sutherland et al., 2010) or a paracellular route (Birkness et al., 1995). To gain more information on the mechanism of barrier traversal across epithelial cells, cells were grown under ALI condition, and we measured TEER at 24 h p.i. as an indication of barrier integrity. To monitor whether infection with the different *N. meningitidis* strains had any influence on barrier integrity we measured TEER at 24h p.i. as well as permeability changes using 70 kDa FITC-dextran as a tracer as described above. Although the different strains successfully traversed the epithelial barrier, there were no notable changes in TEER values (**Figure 4A**). This was also reflected by FITC-dextran measurements. There was no significant difference in the levels of fluorescence determined in the basal chamber of infected cells compared to uninfected control cells (**Figure 4B**).

To further rule out that the expression of tight junction proteins is not altered in response to infection, we conducted qPCR to assess the expression of the tight junction proteins ZO-1 (TJP1) and occludin (OCLN). The expression levels of both proteins remained constant after infection (**Figure 4C**), indicating that neither the disease nor the carrier isolates of both lineages tested in this study altered tight junction expression.

**Figure 5:**
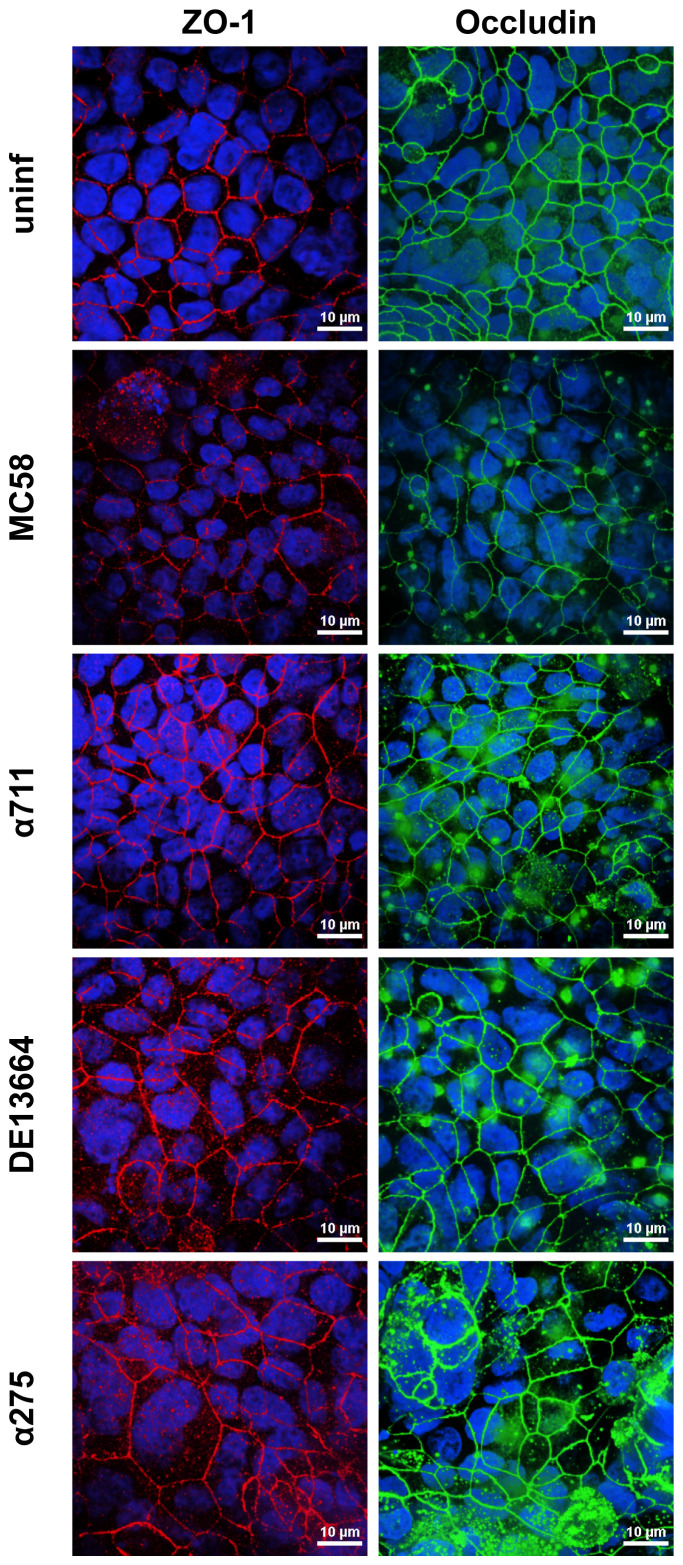
Effects of *N. meningitidis* infection on cell-junction localization in Calu-3 cells. Epithelial cell layers were infected with 1×10^4^ bacteria of meningococcal isolates MenB:cc32 strain MC58, MenB:cc32 strain α711, MenW:cc22 strain DE13664, and MenW:cc22 strain α275 for 24 h or cells were left uninfected as control (uninf). Confocal microscopy images showing tight junction staining for occludin (green), zonula occludens (ZO)-1 (red) in Calu-3 cells grown under ALI conditions. Nuclei staining was performed with DAPI (blue). Scale bars represent 10 µm.

To further assess the effects of *N. meningitidis* on the localization of ZO-1 and occludin, we analyzed Calu-3 cell layers after 24 h of infection with MenB:cc32 and MenW:cc22 strains using confocal microscopy. Here, we observed that neither ZO-1 nor occludin distribution was altered during infection (**Figure 5**), furthermore corroborating that tight junctions remain intact during *N. meningitidis* infection.

The higher efficiency of the disease isolates to transmigrate the barrier might be a result of a higher efficiency to invade into Calu-3 cells. We therefore determined invasion rate for both the disease and carrier isolates of MenB:cc32 and MenW:cc22 strains. The number of recovered intracellular bacteria was significantly lower (2.5× 10^2^cfu/monolayer for MenB:cc32 strain α711 and 4.3 × 10^2^ cfu/monolayer for MenW:cc22 strain α275) compared to the respective disease strains from the same lineage (10.3 × 10^2^ cfu/monolayer for MenB32:cc strain MC58 and 36.3 × 10^2^ cfu/monolayer for MenW:cc22 strain DE13664) (**Figure 4D**). The differences in invasive capacity, however, could not be attributed to the expression of the outer membrane protein Opc, as outlined above (**Supplementary Figure S3**).

**Figure 6:**
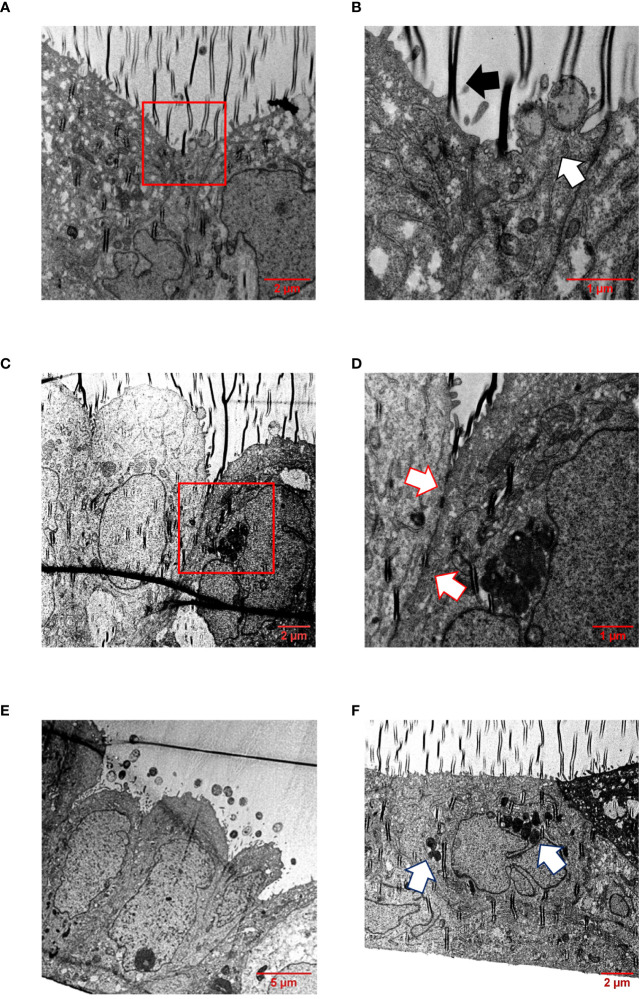
TEM of infected Calu-3 cells grown under ALI conditions. Epithelial cell layers were infected with 1×10^4^ *N. meningitidis* MC58 for 24 h. **(A)** Image shows intimate attachment of meningococci to the cells. Boxed region in **(A)** is enlarged in **(B)**. **(B)** The white arrow indicates adherent bacteria, and the black arrow indicates a folding artefact. **(C)** Intact cell junctional structures. The boxed region in **(C)** is enlarged in **(D)**. **(D)** Enlargement of the region between the cells reveal the presence of major junctional structures (white arrows). **(E)** Monolayers remain intact and appeared undamaged. Image shows bacterial colonies on the apical surface of the epithelial cell layer. **(F)** Bacterial transcytosis across the epithelial cell layer. The white arrows points to bacteria, which are likely situated intracellularly within a vacuole.

In addition, infected Calu-3 monolayers were examined by transmission electron microscopy (TEM) after appropriate preparation of semi-thin sections. TEM pictures showed that all isolates interacted directly with the cells and were able to effectively penetrate the cell layer without damaging the cell-cell junctions (**Figures 6A–E**, **Supplementary Figures S4**-**S6**). The bacteria were found either as single cells (**Supplementary Figure S5F**) or in conglomerates (**Figure 6F**, **Supplementary Figures S4F**, **S6F**), often enclosed within vacuoles, strongly suggesting their intracellular localization (**Figure 6F**), indicating that transcytosis occurs in vacuoles as previously described for human brain microvascular endothelial cells or Calu-3 cells, respectively (Nikulin et al., 2006; Barrile et al., 2015).

### 3.4 Cytokine release from infected Calu-3 cells differs between LLI and ALI cell culture condition and is induced to varying degrees by the different meningococcal isolates

**Figure 7:**
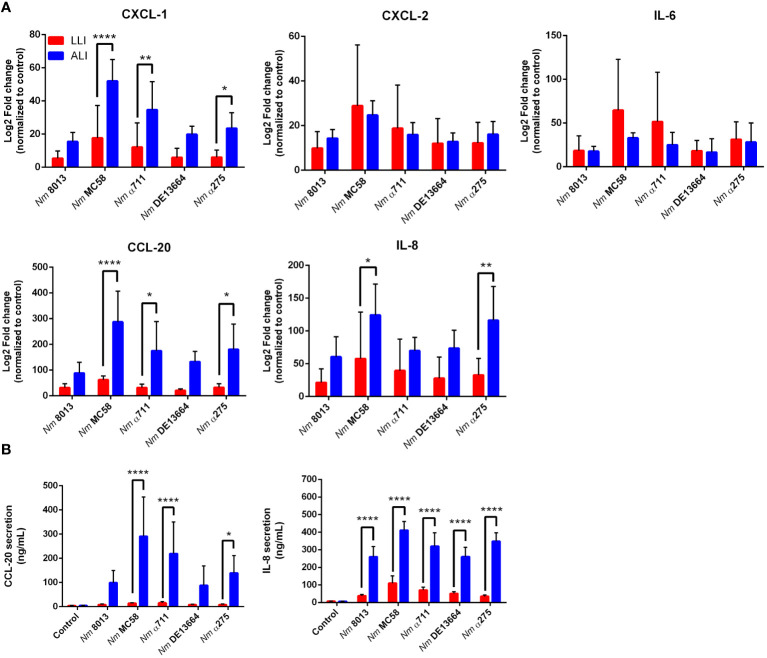
Effects of *N. meningitidis* infection on expression of proinflammatory cytokines/chemokines in Calu-3 cells grown under LLI or ALI conditions. **(A)** Relative expression of CXCL1, CXCL2, CCL20, IL6, and IL8 transcripts in Calu-3 cells grown on 12-Well inserts infected with 4x10^4^ bacteria. Results show the mean ± SD Log2 fold changes, relative to uninfected control cells from three independent experiments performed in duplicates. Data were normalized to 18S rRNA. **(B)** Concentration of CCL-20 and IL-8 measured in the basal chamber at 24 h p.i., determined using ELISA. Data presented as mean ± SD from three independent experiments performed in duplicates. *P < 0.05; **P < 0.01; ****P < 0.0001 by One-way ANOVA.

We next were interested in the cellular immune response after challenging the cells - grown under LLI or ALI conditions - with bacteria and investigated the cytokine responses upon infection. We selected five cytokines, including CXCL-1, CXCL-2, IL-6, IL-8, and CCL-20, and determined their expression levels 24 h after infection using qPCR (**Figure 7A**). MenB:cc32 strain MC58 induced a significant stronger increase in cytokine expression compared to control MenC:cc18 strain 8013 when cells were infected under ALI conditions, CXCL-1 was 3.4-fold higher expressed for MC58-infected cells compared to strain 8013. In general, the cytokine release appeared to be higher from cells grown under ALI conditions compared to LLI conditions. Whereas for CXCL-2 and IL-6, we observed no significant differences in expression levels, we detected a strongly enhanced upregulation for CXCL-1, IL-8, and CCL-20 when cells were grown under ALI culture conditions and infected with the different strains compared to cell grown under LLI conditions. As IL-8 and CCL-20 showed the strongest increase in mRNA expression, we next examined the secretion of these cytokines using commercially available ELISA kits. Calu-3 cells were infected for 24 h, and samples were taken from the basolateral chamber. The ELISA results were consistent with those obtained by qPCR, with the strongest increase observed when cells were grown under ALI conditions and infected with the disease MenB:cc32 strains (**Figure 7B**).

## 4 Discussion

*Neisseria meningitidis* is an effective colonizer of the human nasopharynx, with asymptomatic carriage rates of greater than 25% described in some series of adolescents and young adults (Yazdankhah and Caugant, 2004; Caugant and Maiden, 2009; Christensen et al., 2010). Some genetic lineages have a propensity to transmigrate the respiratory epithelium and cause invasive meningococcal disease (IMD) (Soriani, 2017; Mikucki et al., 2022).

In this study we made use of a Calu-3 air-liquid-interface (ALI) cell culture-based model that induces formation of pseudostratified layers and mucus production and investigated the interaction of carrier and disease *N. meningitidis* isolates belonging to MenB:cc32 and MenW:cc22 with the epithelial barrier. Our data with the hypervirulent lineage MenB:cc32 and the endemic lineage MenW:cc22 showed that all isolates, regardless of the genetic lineage and whether they are a carrier and disease strain, are mainly trapped in the outer mucus layer of the respiratory tract, as recently shown for *N. meningitidis* isolate 8013, a MenC:cc18 strain (Audry et al., 2019). However, the meningococcal isolates tested in our study were able to reach the surface of the epithelial cell and to overcome the mucus layer. Although there were no differences in the number of bacteria found in the cell-attached mucus, we observed a significant difference in the number of bacteria transmigrating the epithelial cell barrier, with the representative disease isolates being more efficient to transmigrate compared to carrier isolates. This could not be attributed to the efficiency of binding to the cell surface, but to the capacity of the disease isolates to invade. Moreover, we found, that the representative meningococcal isolates tested in this study did not damage the epithelial barrier: we did not observe a difference in TEER values under infection, nor was permeability affected. The expression of tight junction proteins was not altered, furthermore supporting the finding that *N. meningitidis* traverses the epithelial barrier by a transcellular route (Sutherland et al., 2010).

The nasopharyngeal respiratory epithelium is lined by pseudostratified columnar ciliated epithelium (Kia'i and Bajaj, 2024). Moreover, the epithelium is coated with mucus, a multicomponent secretion with numerous functions (Sheng and Hasnain, 2022). Mucus creates a physical barrier, provides hydration, and excludes pathogens. It is a reservoir for antimicrobial molecules and antibodies. Respiratory mucus can be separated into the outer and inner (cell-attached) layer. The outer mucus layer is characterized by high viscosity and is composed mainly of MUC5AC and MUC5B with high concentrations of antimicrobials and human defensins (Hughes et al., 2019). In contrast, the inner (cell-attached) mucus, also called periciliary liquid, is a watery layer with low viscosity, allowing cilia to transport the outer mucus toward the oral cavity (Widdicombe, 2002). We first characterized Calu-3 cells grown under ALI conditions following the protocol by Kreft et al. (Kreft et al., 2015) and compared their morphological characteristics with cells grown under LLI cell culture condition. Calu-3 cells grown at ALI conditions showed an improved respiratory phenotype with stratified columnar epithelium, approximately 20-30 µm in height, mucus production, strong expression of CRB3, and TEER values of about > 400 Ω cm^2^, whereas Calu-3 cells grown at LLI conditions observed only approximately 8 µm high, with low expression of mucin and CRB3.

When we infected the Calu-3 cells grown under ALI condition, most bacteria (80–90%) were detected in the outer mucus layer as recently described for the first time in the study by Audry et al. (Audry et al., 2019). Our data with the representative isolates of the hypervirulent lineage MenB:cc32 and the endemic lineage MenW:cc22 demonstrate, that, regardless of the genetic lineage and whether it is a carrier or disease strain, the bacteria are mainly trapped in the outer mucus layer of the respiratory tract. These data underline the finding that *N. meningitidis* acts like a commensal (Audry et al., 2019; Callaghan and Dillard, 2019). However, in contrast to the study conducted by Audry et al., we found that the representative isolates used in this study were able to directly interact with the host cell surface. Moreover, we observed significant differences in their ability to transmigrate Calu-3 cells. The different ability of the disease isolates of both MenB:cc32 and MenW:cc22 to transmigrate the epithelial barrier may be due to variable capacity to adhere or to invade the epithelial cells. Our data revealed that the higher efficiency of the disease isolates to transmigrate the barrier was not attributed to a higher efficiency to reach the host cells surface and attach to the epithelial cell, but to invade. These results are consistent with a previous study from our group, where we tested two disease and two carrier isolates (MenB:cc32 and MenC:cc18 strains) for their ability to adhere to and invade Detroit 562 and NP69 epithelial cell lines and to modulate the cell cycle and found that all isolates adhered equally well to Detroit 562 and NP69 cells, while the carrier isolates were significantly less invasive (von Papen et al., 2016). It is important to note that besides the striking correlation between invasion and transmigration, it cannot be completely ruled out that additional mechanisms are involved that contribute to the lower transmigration of the carrier isolates, such as differences in transmigration speed, intracellular survival, or replication. Additional experiments are necessary to evaluate how these factors might affect transmigration.

The Opc protein is one of the meningococcal factors involved in adhesion to and invasion into host cells: Opc can interact with HSPGs and, via vitronectin and/or fibronectin, enable binding to integrin receptors (Virji et al., 1992, 1994, 1995, 1999; Unkmeir et al., 2002; Sa E. Cunha et al., 2010). A homopolymeric polycytidine (poly-C) tract with variable length, located at the promoter region of the *opcA* gene, regulates the expression of Opc protein at the transcriptional level (Sarkari et al., 1994). Most meningococcal strains contain the *opcA* gene, but strains of certain clonal groups (e.g. ST-11 cc, the cc18 MenC strain 8013) lack the *opcA* gene (Seiler et al., 1996). Here, we found that expression of Opc did not correlate with invasion capacity. MenB:cc32 strain MC58 contains the *opcA* gene with ten Cs in the poly-C stretch, which results in efficient Opc expression; the corresponding carrier isolate α711 contains nine Cs in the poly-C stretch and did not express Opc, suggesting a correlation between Opc expression and invasion. However, MenW:cc22 strain DE13664 contains 8 Cs and therefore does not express Opc, while carrier isolate α275 contains 13 Cs and expresses Opc (**Figure S3**).

The role of other virulence factors of *N. meningitidis* in the interaction with Calu-3 cells grown under either ALI or LLI conditions has been investigated in more detail in other published studies (Sutherland et al., 2010; Audry et al., 2019; Dave et al., 2023). Sutherland et al. used Calu-3 cells grown under LLI conditions and found that successful traversal requires expression of Tfp and capsule (Sutherland et al., 2010). Audry et al. analyzed the expression levels for 13 genes previously shown to be involved in colonization of mucosal surfaces and compared Calu-3 cells cultured under ALI condition with LLI condition. Only four genes, *opaB* and *opaC* (adhesins), *nadA* (adhesin/invasin) (Comanducci et al., 2002), and *fhbp* (factor H binding protein) (Principato et al., 2020) were expressed differently in the two systems, with all four genes being expressed at lower levels when Calu-3 cells were grown under ALI conditions (Audry et al., 2019). In a comparable study by Dave et al., the authors examined adhesion, invasion, and disruption of the monolayers of Calu-3 cells grown under ALI conditions and applied carriage isolates of MenY:cc23, MenY:cc147 and the hypervirulent MenW:cc11 lineage (Dave et al., 2023). A limitation of this study is that the authors did not discriminate between bacteria found in the outer-mucus and cell-associated bacteria. They show variability in the ability of meningococcal isolates to adhere and invade and found that some strains disrupt epithelial monolayers. Especially, all three isolates belonging to MenW:cc11 caused disruption of Calu-3 monolayers. Interestingly, this study demonstrated a dominant role for NadA mediating invasion into Calu-3 cells. Whether NadA plays a role for the differential transmigration efficiency found with lineages MenB:cc32 or MenW:cc22 needs to be elucidated. However, it should be noted that the studies published by Audry et al. or Dave et al. exhibited differences not only in the differentiation time of the ALI culture but also in the performance of the permeability tests. Dave et al., for example, allowed their cells to differentiate for 21 days. Cells were infected for 12 h up to 18 h and FITC-Dextran permeability was measured over a period of 3 h. Interestingly, their data for MC58 confirm our data showing no differences in permeability. The higher FITC-dextran permeability values were observed when cells were infected with MenW:cc11 isolates, which again emphasizes the importance of including meningococci of different clonal lineages in the study.

We also determined the cellular immune response to infection of ALI Calu-3 cells with the various strains. Expression of IL-6, IL-8 (CXCL8), CXCL1, CXCL2, and CCL-20, which are known to be expressed in the respiratory epithelium upon infection (Starner et al., 2003; Shieh et al., 2014; Yamamoto et al., 2014; Glaser et al., 2019), was assessed. All cytokines analyzed were significantly upregulated during infection. When we compared cytokine expression between LLI and ALI, we detected a consistently stronger transcriptional upregulation of IL-8, CXCL1, and CCL20 when Calu-3 cells were grown under ALI conditions. In contrast, we detected a stronger IL-6 expression in Calu-3 cells under LLI conditions, compared to ALI conditions. In addition, we determined secretion of IL-8 and CCL20, moreover, demonstrating significant release of both cytokines from infected ALI Calu-3 cells compared to cells under LLI conditions. These data are contradictory to data published in the study from Audry et al., which showed that three major inflammatory cytokines (IL-6, TNF-α and IL-1β) were secreted significantly less in cells under ALI conditions compared to LLI, suggesting an overall reduction in the inflammatory response under ALI (Audry et al., 2019). One reason for this could be that we have taken the samples from the basal chambers, whereas in the latter study samples were collected from the apical side of the cells. The decision to measure cytokines from the basal side was based on the assumption that immune cells should be recruited from the outside, i.e. from the basal side, to generate an immune response or to increase immune surveillance.

The nasopharynx of healthy individuals is colonized by a wide variety of bacterial genera, including *Streptococcus*, *Corynebacterium*, *Haemophilus*, *Staphyloccocus*, and *Neisseria* sp (Bogaert et al., 2011; Teo et al., 2015; Chen et al., 2022). To efficiently colonize the nasopharyngeal mucosa, the microorganisms must adhere to the mucosal surface, utilize locally available nutrients, and evade the human immune system. Remarkably, some bacteria express glycosidases that degrade the highly abundant glycosylated mucins and use them as carbon sources. Mucin degradation is achieved by a combination of mainly saccharolytic enzymes from the bacteria and proteolytic enzymes from the bacteria and the host (Derrien et al., 2010; Terra et al., 2016). Among the above-mentioned bacterial genera, members of the genera *Streptococcus* (Martins et al., 2016), *Haemophilus* (Davies et al., 1995; Garai et al., 2023) and *Staphylococcus* (Thomas et al., 1993; Shuter et al., 1996) have been shown to bind to mucins. Especially *Pseudomonas aeruginosa* can interact with the thick mucus of the lung of cystic fibrosis patients [reviewed in (Zanin et al., 2016)]. *N. meningitidis* does not express glycosidases to degrade mucins, however, other species like *S. mitis* as a part of the complex microbiota in the respiratory tract hydrolyze mucins and may increase the availability of nutrients that can be metabolized by *N. meningitidis* (Derrien et al., 2010; Audry et al., 2019).

The development of physiologically relevant respiratory epithelium analogues is important for infection studies as these models more accurately reflect the *in vivo* situation. Growing Calu-3 cells on Transwell^®^ inserts [(two-dimensional (2D)] under ALI conditions allows the differentiation of cells into polarized monolayers and formation of pseudostratified layers and mucus production (Kreft et al., 2015; Lee et al., 2023; Silva et al., 2023). However, even the use of 2D cultures on plastic surfaces and filter cultures cannot completely reproduce the microenvironment. The inclusion of 3D *in vitro* models for infection studies could improve our knowledge of the interaction of bacteria with cells of the respiratory tract by providing a more complex 3D environment that better mimics the extracellular matrix surrounding airway tissues, along with the incorporation of co-culture strategies (van der Vaart and Clevers, 2021). 3D *in vitro* models for the respiratory tract are widely engineered for several sorts of different applications from basic research to drug discovery (Soriano et al., 2021) and could be adapted for studies with *N. meningitidis*. As an example, a study by Marrazzo et al. was published recently, who developed an *in vitro* 3D system that reconstructs the human tracheal and bronchial mucosa with the pseudostratified epithelium and underlying stromal tissue (Marrazzo et al., 2016). This model was used to study initial colonization by non-typeable *Haemophilus influenzae* (Marrazzo et al., 2016).

Taken together, our results provide evidence that both disease and carrier isolates of the species *N. meningitidis* irrespective of the genetic lineage are mainly trapped in the outer mucus of the infected respiratory epithelium, however a sufficient proportion of bacteria can reach the surface of host cells and transmigrate across the epithelial barrier. Further exploration of the mechanistic basis that defines the invasive phenotype of disease isolates within *N. meningitidis* strains may improve our understanding of the transition of meningococci from benign commensals to life-threatening pathogens.

## Data availability statement

The datasets presented in this study can be found in online repositories. The names of the repository/repositories and accession number(s) can be found below: https://pubmlst.org/bigsdb?db=pubmlst_neisseria_isolates&page=query, id10183 https://pubmlst.org/bigsdb?db=pubmlst_neisseria_isolates&page=query, id84878.

## Ethics statement

Ethical approval was not required for the studies on humans in accordance with the local legislation and institutional requirements because only commercially available established cell lines were used. Ethical approval was not required for the studies on animals in accordance with the local legislation and institutional requirements because only commercially available established cell lines were used.

## Author contributions

SP: Conceptualization, Data curation, Formal Analysis, Funding acquisition, Investigation, Methodology, Supervision, Visualization, Writing – original draft. KM: Data curation, Formal Analysis, Investigation, Writing – original draft. HC: Data curation, Funding acquisition, Investigation, Methodology, Writing – review & editing. CS: Funding acquisition, Methodology, Resources, Writing – review & editing. AS: Conceptualization, Project administration, Resources, Supervision, Writing – original draft, Funding acquisition, Validation.
