# Supplementary material for: Interaction of Neisseria meningitidis carrier and disease isolates of MenB cc32 and MenW cc22 with epithelial cells of the nasopharyngeal barrier
Source: Front Cell Infect Microbiol. 2024 May 2;14:1389527. doi: 10.3389/fcimb.2024.1389527 (PMC11096551; doi:10.3389/fcimb.2024.1389527)
Supplement: Supplementary file 1 [file DataSheet_1.docx]

Supplementary Material

Interaction of *Neisseria meningitidis* carrier and disease isolates of MenB cc32 and MenW cc22 with epithelial cells of the nasopharyngeal barrier

Simon Peters^1†^, Katherina Mohort^1†^, Heike Claus^1^, Christian Stigloher^2^, Alexandra Schubert-Unkmeir^1*^

^1^Institute for Hygiene and Microbiology, Julius-Maximilian University Wuerzburg, Wuerzburg, Germany

^2^Imaging Core Facility, Biocenter, Julius-Maximilian University Wuerzburg, Wuerzburg, Germany

^†^ These authors contributed equally to this work and share first authorship

*** Correspondence:**Alexandra Schubert-Unkmeir

aunkmeir@hygiene.uni-wuerzburg.de

Keywords: *Neisseria meningitidis*, clonal complexes, transmigration, air-liquid-interface, epithelial barrier

**Material and Methods**

**PCR and Sequencing**

For sequencing*, opcA* promotor region was amplified and sequenced from single colonies by PCR using forward primer RA3 (5´-CATCTCAAGTCTCGTCATTCC-3´) and reverse primer RA4 (5´-AGCCTGTGTAAAGATCGATAC-3´). Nucleotide sequence data were analyzed with the Lasergene^®^ software (DNASTAR, Madison, WI).

**Western blot**

Bacterial samples for western blot were prepared by lysis of 1x10^9^ bacteria in lysis buffer (0.05 M Tris-HCl, 50 mM EDTA, 100 µg/ml lysozyme) for 1 h while shaking. Further lysis was achieved by three freeze and thaw cycles. Debris were removed by centrifugation at 1,200 x g for 10 min. at 4° C and protein concentration was determined by BCA-assay. Equal protein concentrations were loaded and separated on a 12 % SDS gel, followed by protein blotting onto a nitrocellulose membrane. Membrane was blocked with 5 % skim milk in TBS-T for 1 h and primary antibodies were applied in the same buffer: mouse anti-PilE (SM1, 1:4,000, provided by M. Virji) mouse anti-Opc (1:1,000, provided by M. Virji) and mouse anti-Opa (1:1,000). After overnight incubation at 4°C membranes were washed three times with TBS-T prior to the addition of the secondary HRP conjugated anti-mouse antibody (in TBS-T + 5 % skim milk, 1:10,000) for 1 h at room temperature. After three washing steps, membrane was overlaid with ECL substrate (BioRad) and proteins were visualized with the ChemiDoc MP (Biorad).

**Bacterial growth**

Bacteria from liquid cultures were centrifuged at 1,100 g for 5 min and resuspended in Calu-3 medium. Afterwards, bacteria were inoculated to an OD_600_ of 0.1 in 10 ml fresh medium with, or without, 0.1 % N-acetylcystein (N-ACC) and incubated with shaking (200 rpm) at 37 °C. Growth was determined by OD_600_ measurement at 0 min, 30 min and 60 min.


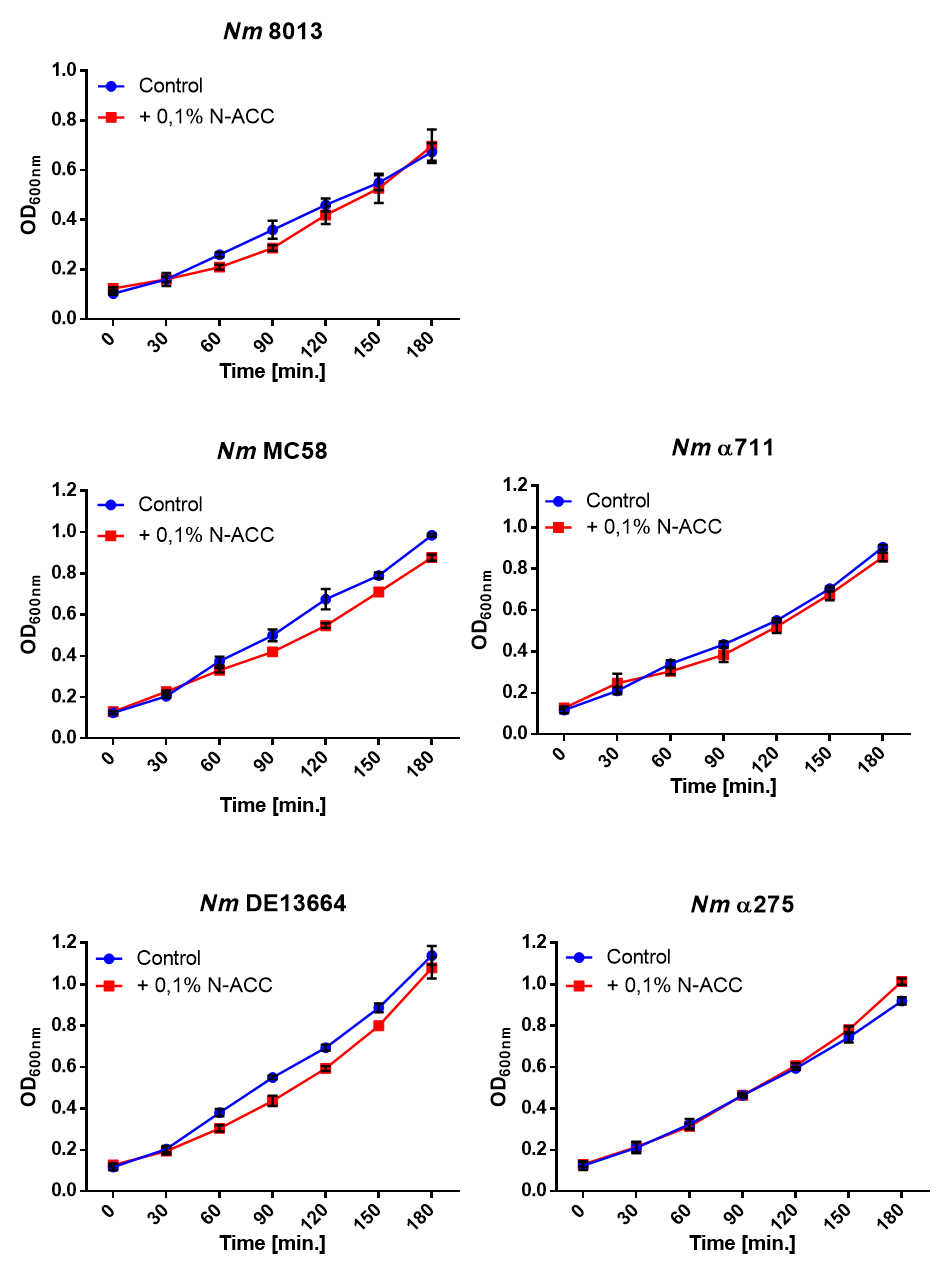


**Supplementary Figure 1.**

Growth analysis of strains tested in this study treated with 0.1 % N-acetylcystein (N-ACC). Bacteria were adjusted to an OD600 of 0.1 in 10 ml of Calu-3 medium with, or without, N-ACC. OD600 was measured at 0 min, 30 min, and 60 min.

**CRB**

**
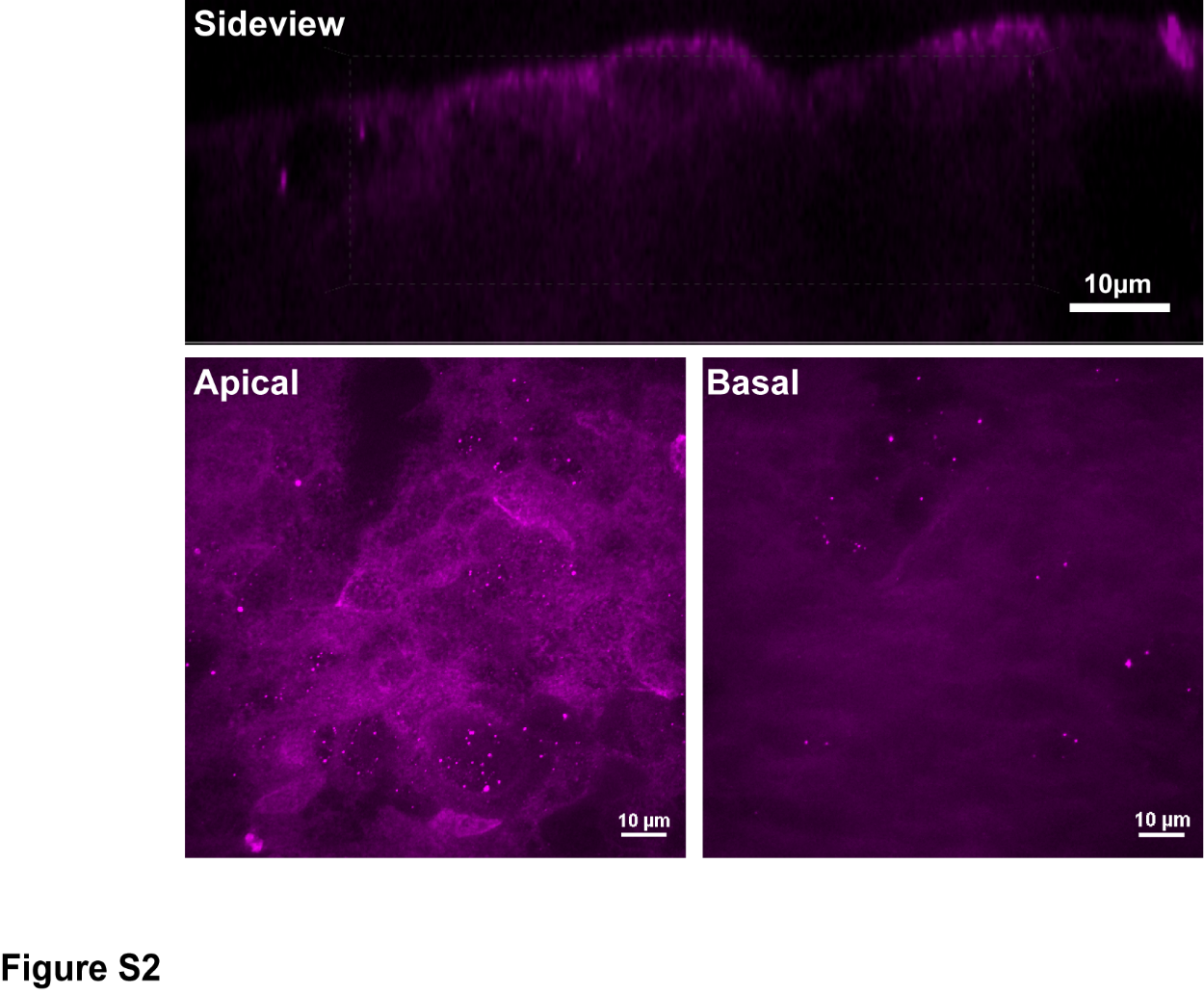
**

**Supplementary Figure 2.**


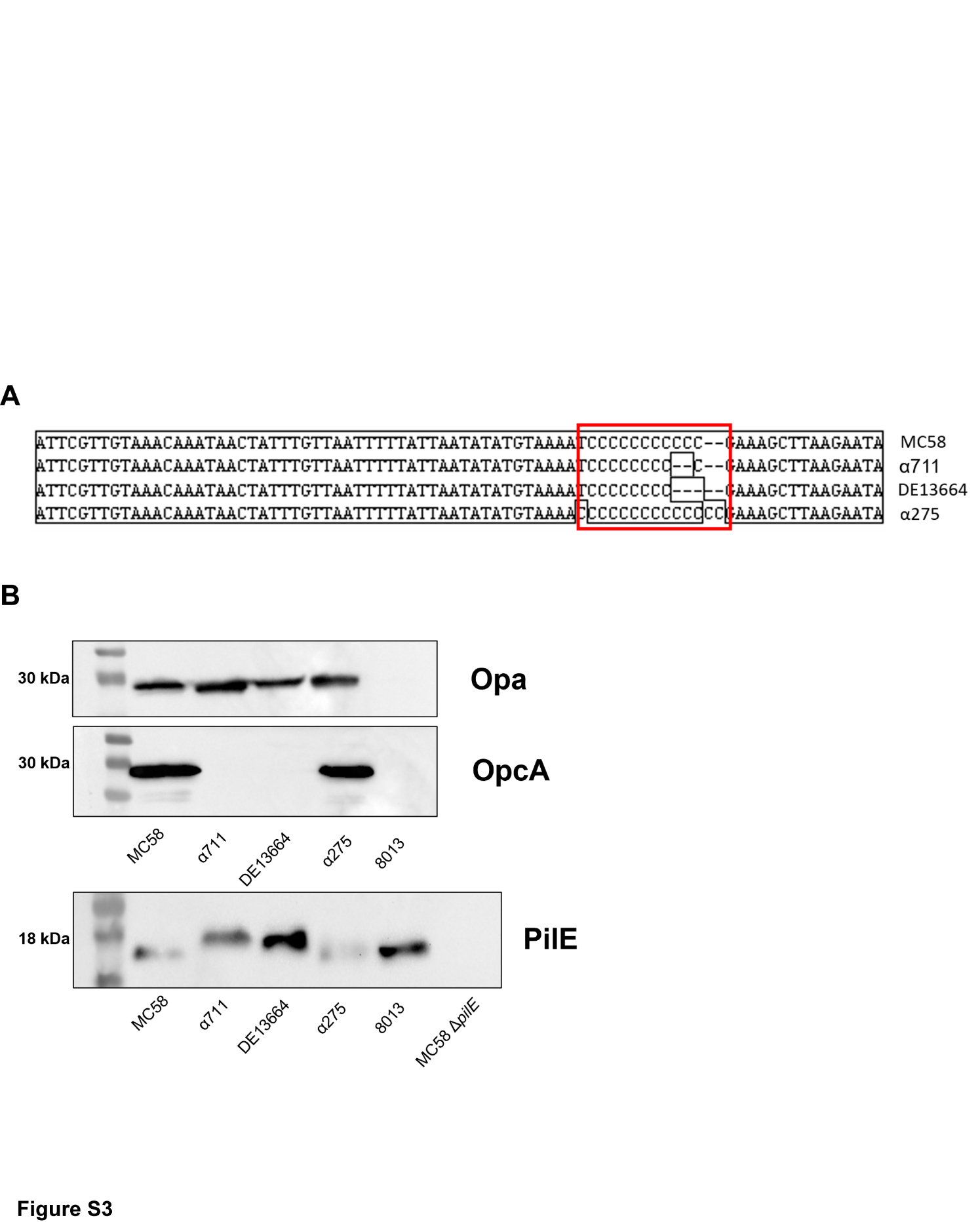


**Supplementary Figure 3.**

(A) Sequence alignment of the *opcA* promotor region of *N. meningitidis* MC58 (reference strain), α711, DE13664, and α275. Tract of contiguous C residues (poly-C) is shown. (B) Western blot analysis of whole cell lysates from *N. meningitidis* strain 8013/clone 12 (2), MC58 (3), α711 (4), DE13664 (5), α275 (6), MW Marker (1) using Opc-specific Mab B306, pan-Opa antibody 4B12/C11, and SM1 Mab recognizing class I pili.


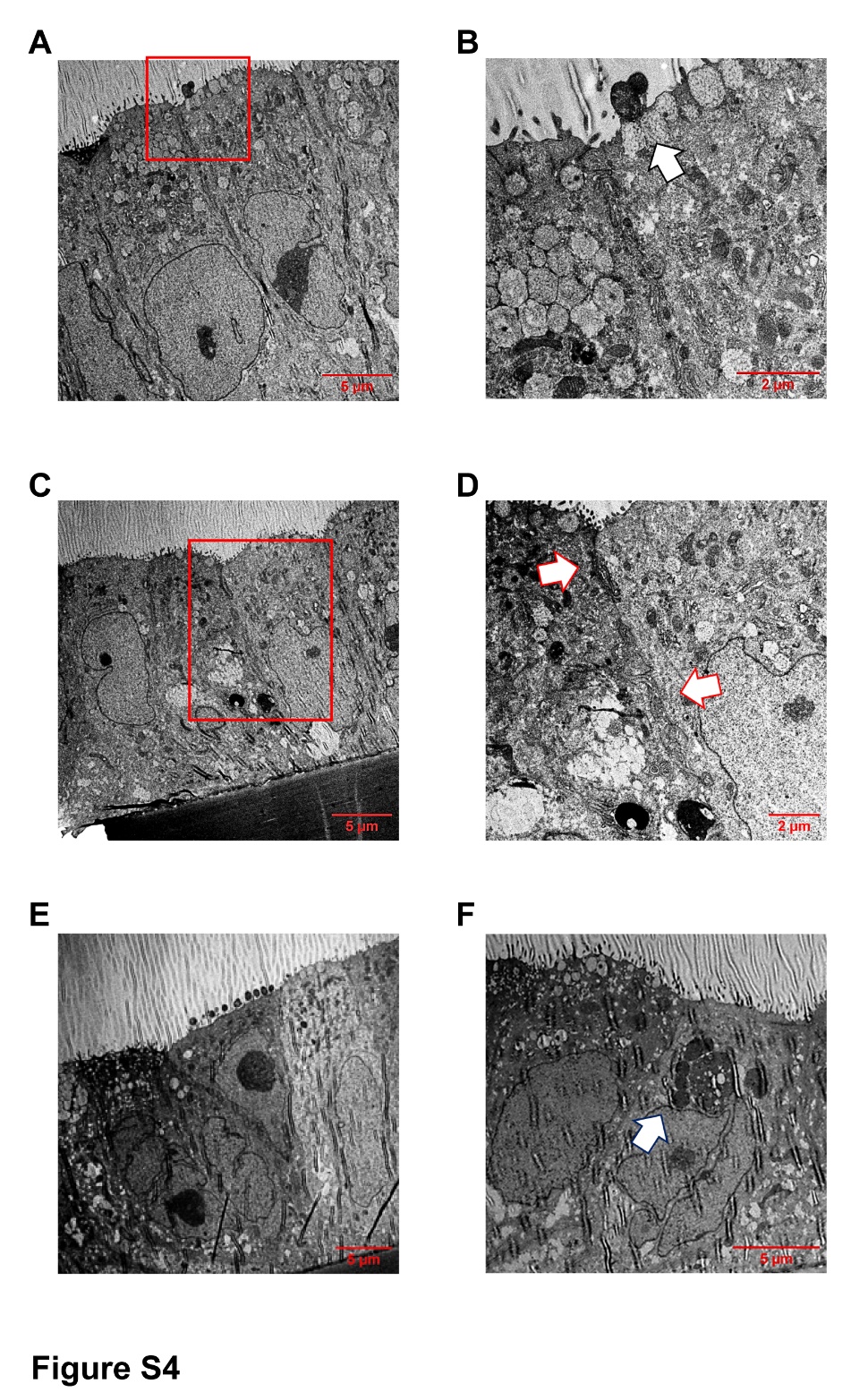


**Supplementary Figure 4.** TEM of infected Calu-3 cells grown under ALI conditions. Epithelial cell layers were infected with 1×10^4^ *N. meningitidis* α711 for 24 h. (A) Image shows intimate attachment of meningococci to the cells. Boxed region in panel A is enlarged in panel B. (B) The white arrow indicates adherent bacteria. (C) Intact cell junctional structures. The boxed region in panel C is enlarged in panel D. (D) Enlargement of the region between the cells reveal the presence of junctional structures (white arrows) (E) Monolayers remain intact and appeared undamaged. Image shows bacterial colonies on the apical surface of the epithelial cell layer. (F) Bacterial transcytosis across the epithelial cell layer. The white arrow points to bacteria, which are likely situated intracellularly within a vacuole.


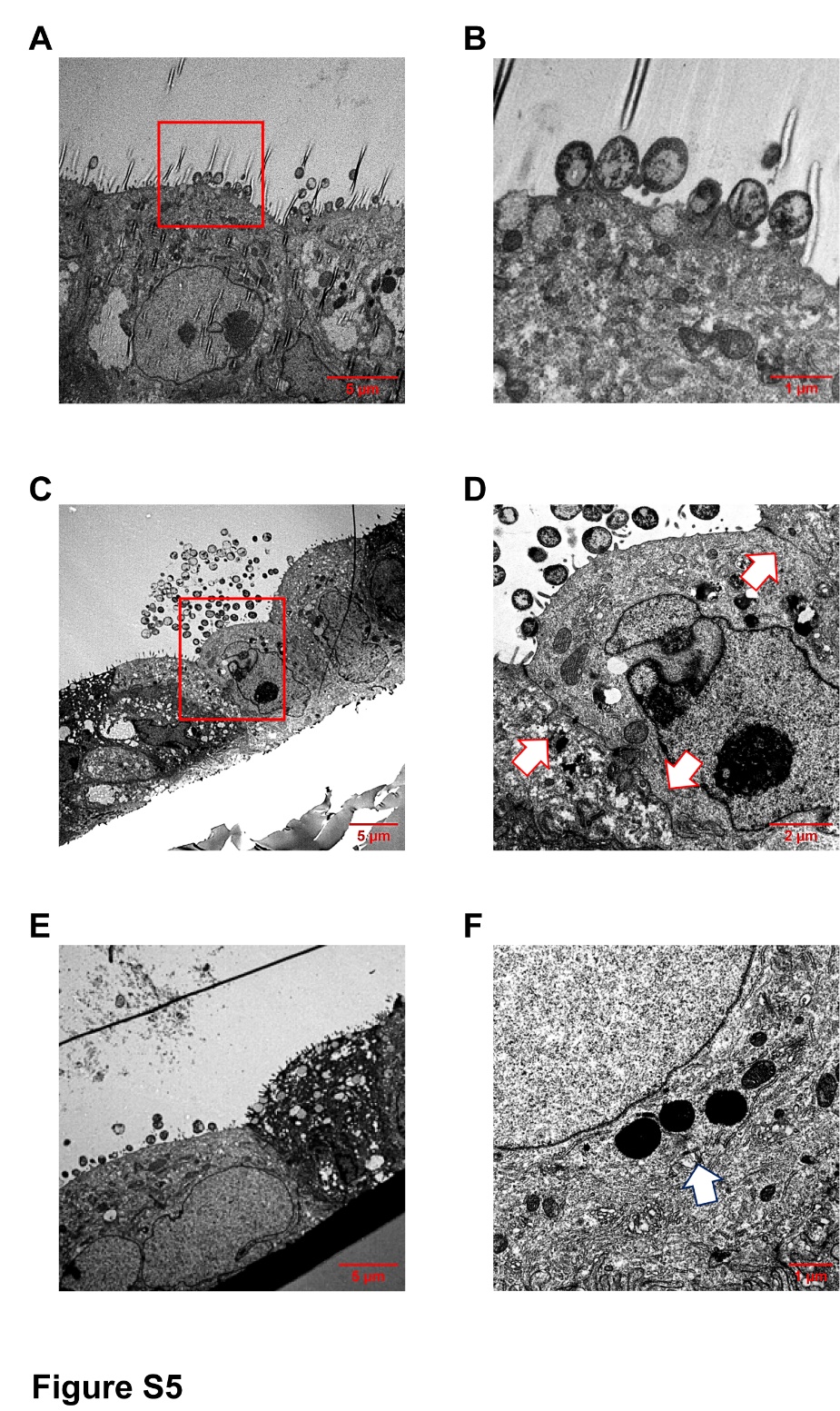


**Supplementary Figure 5.** TEM of infected Calu-3 cells grown under ALI conditions. Epithelial cell layers were infected with 1×10^4^ *N. meningitidis* DE13664 for 24 h. (A) Image shows intimate attachment of meningococci to the cells. Boxed region in panel A is enlarged in panel B. (C) Intact cell junctional structures. The boxed region in panel C is enlarged in panel D. (D) Enlargement of the region between the cells reveal the presence of junctional structures (white arrows) (E) Monolayers remain intact and appeared undamaged. Image shows bacterial colonies on the apical surface of the epithelial cell layer. (F) Bacterial transcytosis across the epithelial cell layer. The white arrow points to bacteria, which are likely situated intracellularly within a vacuole.


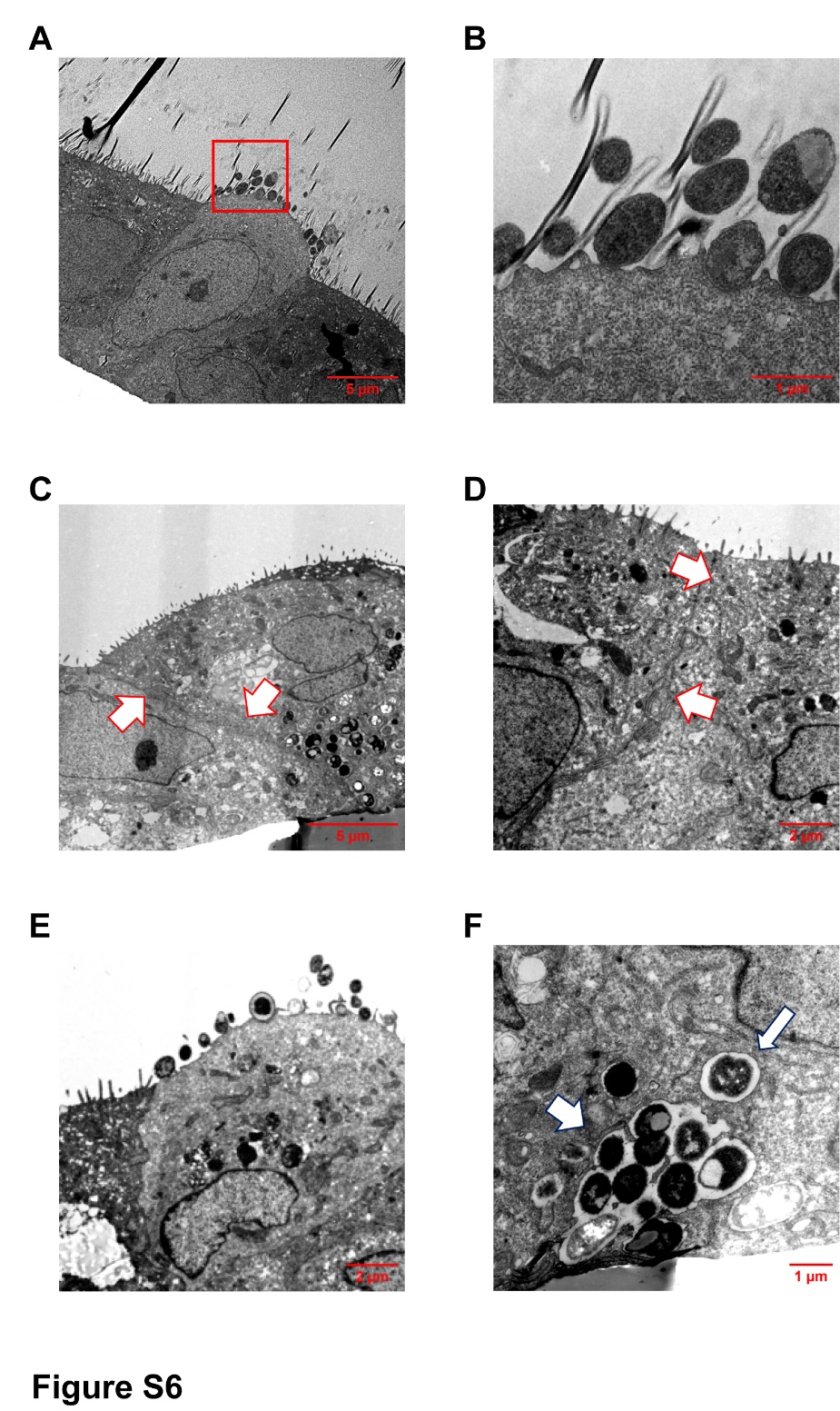


**Supplementary Figure 6.** TEM of infected Calu-3 cells grown under ALI conditions. Epithelial cell layers were infected with 1×10^4^ *N. meningitidis* α275 for 24 h. (A) Image shows intimate attachment of meningococci to the cells. Boxed region in panel A is enlarged in panel B. (C, D) Intact cell junctional structures. The arrows indicate the presence of junctional structures (white arrows) (E) Monolayers remain intact and appeared undamaged. Image shows bacterial colonies on the apical surface of the epithelial cell layer. (F) Bacterial transcytosis across the epithelial cell layer. The thin white arrow points to bacteria, which are likely situated intracellularly within a vacuole. The bold white arrow denotes a cluster of bacteria, potentially enclosed within vacuoles, or alternatively, positioned at the intercellular junction, allowing direct contact with the extracellular space.
